# Identification and HLA-Tetramer-Validation of Human CD4^+^ and CD8^+^ T Cell Responses against HCMV Proteins IE1 and IE2

**DOI:** 10.1371/journal.pone.0094892

**Published:** 2014-04-23

**Authors:** Peter Braendstrup, Bo Kok Mortensen, Sune Justesen, Thomas Østerby, Michael Rasmussen, Andreas Martin Hansen, Claus Bohn Christiansen, Morten Bagge Hansen, Morten Nielsen, Lars Vindeløv, Søren Buus, Anette Stryhn

**Affiliations:** 1 Laboratory of Experimental Immunology, Faculty of Health Sciences, University of Copenhagen, Copenhagen, Denmark; 2 The Allogeneic Hematopoietic Cell Transplantation Laboratory, Department of Hematology, Rigshospitalet, Copenhagen University Hospital, Copenhagen, Denmark; 3 Department of Clinical Microbiology, Rigshospitalet, Copenhagen University Hospital, Copenhagen, Denmark; 4 Department of Clinical Immunology, Rigshospitalet, Copenhagen University Hospital, Copenhagen, Denmark; 5 Center for Biological Sequence Analysis, Department of Systems Biology, Technical University of Denmark, Lyngby, Denmark and Instituto de Investigaciones Biotecnológicas, Universidad de San Martín, San Martín, Buenos Aires, Argentina; University of Regensburg, Germany

## Abstract

Human cytomegalovirus (HCMV) is an important human pathogen. It is a leading cause of congenital infection and a leading infectious threat to recipients of solid organ transplants as well as of allogeneic hematopoietic cell transplants. Moreover, it has recently been suggested that HCMV may promote tumor development. Both CD4^+^ and CD8^+^ T cell responses are important for long-term control of the virus, and adoptive transfer of HCMV-specific T cells has led to protection from reactivation and HCMV disease. Identification of HCMV-specific T cell epitopes has primarily focused on CD8^+^ T cell responses against the pp65 phosphoprotein. In this study, we have focused on CD4^+^ and CD8^+^ T cell responses against the immediate early 1 and 2 proteins (IE1 and IE2). Using overlapping peptides spanning the entire IE1 and IE2 sequences, peripheral blood mononuclear cells from 16 healthy, HLA-typed, donors were screened by *ex vivo* IFN-γ ELISpot and *in vitro* intracellular cytokine secretion assays. The specificities of CD4^+^ and CD8^+^ T cell responses were identified and validated by HLA class II and I tetramers, respectively. Eighty-one CD4^+^ and 44 CD8^+^ T cell responses were identified representing at least seven different CD4 epitopes and 14 CD8 epitopes restricted by seven and 11 different HLA class II and I molecules, respectively, in total covering 91 and 98% of the Caucasian population, respectively. Presented in the context of several different HLA class II molecules, two epitope areas in IE1 and IE2 were recognized in about half of the analyzed donors. These data may be used to design a versatile anti-HCMV vaccine and/or immunotherapy strategy.

## Introduction

Human cytomegalovirus (HCMV) is a member of the ubiquitous *Betaherpesvirinae* subfamily, which infects 50–100% of the adult population[1]. In healthy immunocompetent individuals, HCMV establishes a life-long asymptomatic latent infection where intermittent sub-clinical reactivations are successfully controlled by the immune system. In contrast, in individuals without adequate immune-mediated control, HCMV infection results in considerable morbidity and even mortality. This includes recipients of solid organ transplants (SOT) or allogeneic-hematopoietic cell transplants (allo-HCT) that are given immunosuppressive treatment where HCMV is one of the most frequent and clinically relevant infectious complications[2], [3], [4], [5], [6]. Indeed, most immunosuppressive strategies include a component that closely monitors HCMV infection allowing immediate preemptive anti-viral therapy should HCMV reactivation be detected. Another important area of HCMV-mediated pathogenicity is that of congenital HCMV infection. It is the most frequent and important congenital infection where it can lead to severe developmental abnormalities and fetal death[7]. Lastly, HCMV has been implicated in various human cancers[8] with immediate early (IE) proteins possibly playing a key role in promoting carcinogenesis[9]. Thus, a recent study showed significantly improved survival of glioblastoma patients receiving valganciclovir in combination with conventional chemotherapy as compared to patients only receiving chemotherapy[10]. Overall, HCMV is a significant health burden[11].

How to prevent and/or treat HCMV infection is therefore a highly relevant medical issue. Current anti-viral drugs such as ganciclovir and foscarnet have serious adverse effects such as impaired hematopoietic recovery and nephrotoxicity[12]. Thus, there is a need for safer and more efficient alternatives. All components of the adaptive immune system, B cells, CD4^+^ T helper cells (Th), and CD8^+^ cytotoxic T cells (CTLs)[2], [13], [14], [15], are involved in generating and maintaining anti-HCMV immunity, and it is believed that vaccination and/or immunotherapy may provide efficient prevention and/or treatment without side effects[16], [17], [18]. In particular, trials with adoptive T cell transfer of HCMV-specific T cells to recipients of allo-HCT have been encouraging[19], [20], [21], [22]. Thus, adoptive transfer of CD8^+^ CTLs has been reported to restore cellular immunity against HCMV in human patients (e.g. [19], [23]) as well as in a murine model of cytomegalovirus[24]. From studies of the murine immune system, it is known that CD4^+^ Th cell activity is important for maintenance of immunological memory[25], [26]. That a similar need for CD4^+^ Th exists in protection against HCMV is suggested by studies showing that durable HCMV-specific T cell immunity depends on the presence of HCMV-specific CD4^+^ T cells [20], [27], [28], by observations that specific CD8^+^ T cells can clear ongoing HCMV infection, but not establish lasting immunity[27], [28], and by the association of suppression of CD4^+^ T cell responses and HCMV disease in HIV patients[29]. Thus, trials of adoptive T cell therapy should include both CD4^+^ and CD8^+^ T cells specific for HCMV[17].

A particularly promising approach involved the use of a single peptide-HLA class I tetramer to obtain an anti-HCMV reactive CD8^+^ T cell preparation of a single specificity from appropriate HCMV-seropositive donors[19]. Immediately after preparation, these mono-specific CD8^+^ CTLs were transferred to allo-HCT patients, where they proliferated and showed *in vivo* activity. HCMV viremia was reduced in all nine recipients and cleared in eight of them. No side effects were observed. This suggests that simple direct epitope-specific adoptive T cell transfer could afford efficient and safe HCMV protection. In fact, a current phase 2 trial is evaluating a similar approach to select HCMV-specific T cells, with the aim of preventing reactivation and disease[30]. It is a reasonable assumption that a multi-epitope approach would be even more efficient in protecting the host from uncontrolled HCMV replication since broadening immune reactions to a larger repertoire of known HCMV-specific T cell epitopes should multiply and diversify the immune response and stand a better chance of controlling a virus at any phase of its life cycle thereby minimizing the risk of viral escape. Furthermore, including CD4^+^ Th cells recognizing one or more epitopes should contribute towards maintaining immune memory and protection. Thus, using a multi-epitope approach should be an advantage[31], [32], [33], and should be instrumental in enabling specific adoptive T cell transfer to most, if not all, immunocompromised patients in need of anti-HCMV prevention and/or therapy.

The purpose of this report is to extend our knowledge of frequently recognized (i.e. dominant) anti-HCMV-specific CD4 and CD8 epitopes. To this end we have systematically examined cellular immune responses against proteins encoded by the IE regulatory genes. These are essential for viral gene expression and replication[34]. Of particular interest here, IE, early, and late class genes being expressed in temporal order defines a replicative cycle. Thus, the first wave is characterized by the transcription of IE genes, and T cells recognizing IE epitopes should target the first gene products expressed during reactivation. Interestingly, a study of heart and lung transplant recipients suggested that T cells recognizing IE gene products may be crucial for virus control, as CD8^+^ T cell reactivity against IE1 protein, but not against the otherwise immunodominant 65-kDa phosphoprotein (pp65), correlated with protection from HCMV disease[35].

The most extensively studied IE proteins, the 72 kDa-IE1 protein (IE1) and the 86 kDa-IE2 protein (IE2), are two of several IE gene products; the IE2 gene is indispensable for viral replication, and deletion of the IE1 gene reduces viral replication[36], [37]. Both proteins have previously been found to be highly immunogenic[15]. In the present study we used overlapping peptides spanning the entire IE1 and IE2 sequences and a combination of bioinformatics, immunochemistry, and cellular immunology to identify IE1- and IE2-specific T cell responses in 16 healthy HCMV-reactive donors. The ability to induce CD4^+^ and CD8^+^ T cell responses *ex vivo* and after an *in vitro* culture was evaluated with combinations of ELISpot and flow cytometric intracellular cytokine secretion assays (ICS). Further epitope characterization and restriction validation of CD4^+^ and CD8^+^ T cell responses was done using bioinformatics prediction tools, peptide-HLA binding analysis, and HLA class I and II tetramer staining. Eighty-one CD4^+^ and 44 CD8^+^ T cell responses were identified in the 16 donors, and in many cases the underlying peptide-specific, HLA-restricted reactivity of these responses were validated with appropriate peptide-HLA class I or II tetramers. Many of these specificities were recognized in several different donors, and may therefore serve as broadly relevant targets for immune-mediated prevention and/or therapy of HCMV infection.

## Materials and Methods

### Donors (and Ethics Statement)

The study of donor immune responses was approved at the National University Hospital of Copenhagen by “The Committees on Biomedical Research Ethics of the Capital Region” (Danish: “De Videnskabsetiske Komiteer for Region Hovedstaden”) (RH-3-CT5604) with informed written consent.

Buffy coats were obtained from 16 healthy Danish blood donors (age 35–65 years). Peripheral blood mononuclear cells (PBMC) were isolated by density gradient centrifugation using Ficoll-Paque Plus (GE Healthcare Europe, Brøndby, Denmark), and stored until use at −150°C.

Chromosomal DNA was isolated from all donors and typed for HLA-A/B/C and HLA-DR/DQ/DP using Sequence Based Typing (Genome Diagnostics, Utrecht, the Netherlands).

### Peptides

The primary sequences of the 412 amino acid long IE1 and the 580 amino acid long IE2 from the HCMV isolate AD169 were obtained from the UniProt database (www.UniProt.org, accession numbers P13202 (IE1) and P19893 (IE2)). Fifteen amino acid long peptides overlapping by 10 amino acids spanning the entire IE1 and IE2 protein, a total of 187 peptides (78 IE1 peptides and 109 IE2 peptides), were synthesized. Note that the initial 85 amino acids of IE1 and IE2, corresponding to fourteen 15mer peptides, were identical and only represented once in the IE2 peptide pool. The peptides were used either individually or in pools of IE1 peptides or IE2 peptides.

Peptides were synthesized by standard 9-fluorenylmethyloxycarbonyl chemistry and purified by reversed-phase high-performance liquid chromatography (purity at least 80%, usually >95%) (Schafer-N, Copenhagen, Denmark). In addition, pools of 15-amino acid long overlapping peptides spanning the entire pp65 protein (strain AD169) were obtained from JPT Peptide Technologies, Berlin, Germany.

### *Ex vivo* Interferon-γ ELISpot assay

An interferon-γ (IFN-γ) ELISpot assay was performed as previously described[38]. Briefly, PBMCs were thawed, resuspended in Xvivo15 (Lonza) supplemented with 5% AB serum (Invitrogen) – “complete medium”, and incubated at 2–5×10^5^ cells/well in an anti-IFN-γ (mAb1-D1K, MabTech, Nacka Strand, Sweden)-coated ELISpot plate (MAHAS4510, Merck Milipore, Billerica, USA) for 18–24 h in the presence or absence of peptides at a final concentration of 1 µM. As positive controls, cells were stimulated with Staphylococcal enteroxin B (SEB, Sigma Aldrich, St. Louis, USA) at a final concentration of 1 µg/ml. Two wells without peptide served as negative control. Plate-bound IFN-γ was detected with biotinylated antihuman IFN-γ (mAb 7-B6-1, MabTech, Nacka Strand, Sweden) and developed by addition of streptavidin conjugated alkaline phosphatase (Streptavidin ALP, MabTech, Nacka Strand, Sweden) and substrate (AP Conjugate substrate, Bio-Rad, Hercules, USA). Analysis was done using ImmunoSpot 5.0.9 software (C.T.L., Shaker Heights, USA). Observed background range was 0–10 spot forming units (SFU)/10^6^ PBMC (average 3 SFU/10^6^ PBMC). As others have noted, there is no consensus on the definition of a positive response in ELISpot and other assays employed to detect antigen-specific T cells and antigen-specific T cell responses[39]. We chose 25 SFU/10^6^ PBMC (negative control subtracted) as a threshold for positive responses. Peptides eliciting these responses were selected for subsequent *in vitro* culture.

### Cell cultures

#### PBMCs

PBMCs were incubated overnight with 1 µM of peptide in complete medium. At day 2 the cells were harvested, washed, and plated in new wells with 50 U/ml IL-2. Fresh medium and IL-2 were added every second day. From day 6, IL-15 was added every second day. The cells were harvested for analysis at day 12–14.

#### Dendritic cells (DCs)

In some cases the analysis of HLA restriction was performed using DCs and HLA-matched allo-presentation. Briefly, DCs were generated from adherent cells after 1.5 h incubation of PBMCs at 37°C, 5% CO2. The adherent cells were cultured for 8–11 days in complete medium supplemented with granulocyte-monocyte colony-stimulating factor (250 U/ml) and IL-4 (500 U/ml). Fresh medium and cytokines were added every third day. The DCs were activated with tumor necrosis factor-α (TNF-α) (10 ng/ml), IL-6 (20 ng/ml), IL-1β (5 ng/ml) and prostaglandin E2 (PGE2) (1 µg/ml) 48 h before use. All cytokines were purchased from Peprotech, Germany, except for PGE2, which was purchased from Sigma-Aldrich, Germany.

### Intracellular cytokine secretion assay (ICS)

#### Ex vivo

Thawed PBMCs were resuspended in complete medium and aliquoted at 1×106 cells/well in 96-well round bottom microtiter plates. Cells were stimulated with or without various different peptides and peptide pools (1 µM of each peptide) and costimulatory CD28/49d antibody (1 µg/ml) (Becton Dickinson) for 6 h at 37°C, 5% CO2. Brefeldin A (Sigma-Aldrich) was present for the last 5 h of incubation.

#### *In vitro* culture

In vitro cultured PBMCs were harvested, washed, resuspended in complete medium, and aliquoted at 2–4×105 cells/well. The cells were incubated with relevant single peptide (1 µM) for 4 h at 37°C, 5% CO2. Brefeldin A was present for the last 3 h of incubation.

#### ICS

The cells were subsequently incubated with EDTA (final concentration 1.4 mM) at RT. Afterwards the cells were permeabilized (Becton Dickinson Permeabilizing solution 2) and stained with anti-CD3–allophycocyanin(APC)/Cy7, anti-CD4-peridinin chlorophyll(PerCp), anti-CD8-APC, anti-CD69-R-phycoerythin(PE), and anti-IFN-γ-fluorescein isothiocyanate(FITC) (Biolegend, San Diego, USA). Finally the cells were fixed in 1% formaldehyde and analyzed by flow cytometry on LSRII (BD Biosciences).

#### HLA-matched allo-presentation analysis

Autologous or HLA-matched allogeneic DCs were pulsed with peptides at a final concentration of 0.1–0.3 µM and incubated for 90 min at 37°C, then washed and irradiated (2000 rad). T cells were added, incubated for 4 h, and analyzed by the protocol for ICS assay described above.

### Tetramer staining

#### HLA class I tetramers

HLA class I tetramers were produced as previously described [40]. Briefly, biotinylated recombinant HLA class I heavy chains were diluted into a reaction buffer containing 50 mM tris-maleate pH 6.6, 0.1% Pluronic F86 NF (BASF, a surfactant compatible with cellular use), an excess of β2-microglobulin (β2m) and peptide, and incubated for 48 h at 18oC. To tetramerize the resulting peptide-HLA class I monomers, Streptavidin-PE or Streptavidin-APC (Biolegend, San Diego, USA) was sequentially added over 60 min at a 1∶4 molar ratio of Streptavidin to peptide-HLA-I monomers. PBMCs were resuspended in 25 µl PE- and APC-conjugated tetramer and incubated for 20 min at RT followed by 30 min incubation with anti-CD3-Pacific blue, anti-CD4-APC/Cy7, and anti-CD8-PerCP antibody (Biolegend, San Diego, USA).

#### HLA class II tetramers

HLA class II tetramers were produced as previously described[41]. Briefly, recombinant HLA-DR α- and β-chains were folded in the presence of a C-terminally hexahistidine(H_6_)-tagged version of the peptide in question. The peptide-HLA class II complexes were subsequently purified on a Ni^2+^ charged iminodiacetic acid column. The resulting monomers were tetramerized with PE- or APC-conjugated Streptavidin as described for the HLA class I tetramer above. In vitro cultured PBMCs were incubated with PE- and APC-conjugated HLA class II tetramers for 1 h at 37°C, 5% CO_2_. The cells were washed and subsequently stained with anti-CD3-Pacific blue and anti-CD4-PerCP antibody (Biolegend, San Diego, USA) for 30 min. All tetramer-stained cells were analyzed by flow cytometry on LSRII (BD Biosciences).

### Prediction of epitope and HLA-restriction of T cell responses

#### HLA class I-restricted CD8^+^ T cell responses

For each donor, all 15mer peptides eliciting a CD8^+^ T cell response were submitted to our bioinformatics predictor, HLArestrictor (www.cbs.dtu.dk/services/HLArestrictor/), which predicts the optimal epitopes that could bind to any of the donors HLA-A, -B, or-C molecules of the donor in question [42].

#### HLA class II-restricted CD8^+^ T cell responses

For each donor, all 15mer peptides eliciting a CD4^+^ T cell response were submitted to our bioinformatics predictor, NetMHCIIpan (www.cbs.dtu.dk/services/NetMHCIIpan/), which predicts the binding of the 15mer peptide to all of the HLA-DR class II molecules available to the donor in question, as well as the peptide core sequence interacting with the HLA class II molecule [43]. NetMHCIIpan is currently limited to predicting peptide binding of HLA-DR molecules.

### Biochemical peptide HLA class I and HLA class II binding assays

#### Peptide binding to HLA class I and II

Peptide-HLA class I and II binding affinities were determined as previously described [44], [45]. For HLA class I, denatured and purified recombinant HLA class I heavy chains were diluted into a refolding buffer (tris-maleate buffer, pH 6.6) containing β_2_m and graded concentrations of the test peptide, and incubated for 48 h at 18°C to allow for equilibrium to be reached. For HLA class II, denatured and purified recombinant HLA class II α- and β-chains were diluted into a refolding buffer containing graded concentrations of the test peptide, and incubated for 48 h at 18°C to allow for equilibrium to be reached.

Complex formation was detected using a proximity-based Luminescent Oxygen Channeling Immunoassay assay and the peptide concentration leading to half-saturation (ED_50_) was determined as previously described [44], [45]. Under the limited receptor concentrations used here, the ED_50_ reflects the affinity of the interaction.

### Peptide-HLA class I Stability Measurements

The stability of peptide-HLA class I complexes was measured using ^125^I radiolabelled β_2_m in a scintillation proximity assay as previously described [46]. Briefly, recombinant, biotinylated HLA class I heavy chains were diluted into a refolding buffer containing the test peptide and trace amounts of ^125^I radiolabeled β_2_m, and allowed to refold at 18°C for 24 h in a Streptavidin-coated scintillation microplate (Flashplate PLUS, Perkin Elmer, Boston, MA). Dissociation was initiated by adding excess of unlabeled β_2_m and placing the microplate in a scintillation counter (TopCount NXT, Packard) adjusted to 37°C. The microplate was read continuously for 24 h thereby allowing the dissociation of radiolabeled β_2_m to be determined.

## Results

### Exemplifying the epitope screening strategy

Fifteen amino acid long peptides overlapping by 10 amino acids were used to scan through the entire HCMV-derived IE1 and IE2 protein sequences. This choice of peptide size and overlap was aimed at optimizing the chances of detecting both CD4^+^ T cell responses, which preferably recognize longer peptides, and CD8^+^ T cell responses, which preferably recognize shorter derivatives generated during the cell culture[47]. Using functional T cell read-outs to identify peptides of interest, this should result in a complete search for CD4^+^ and CD8^+^ T cell epitopes.

Initially, PBMCs from healthy donors were screened for HCMV-specific CD4^+^ and CD8^+^ T cell responses by an *ex vivo* ICS assay using pools of peptides derived from the dominant HCMV proteins pp65, IE1, or IE2 as targets. Sixteen donors who showed positive CD4^+^ and/or CD8^+^ T cell responses against at least one of these peptide pools were selected for this study (Table 1). In terms of overall cellular HCMV responsiveness (i.e. delivering either a CD4^+^ or CD8^+^ T cell response); three of the 16 (19%) donors recognized one of the three antigens; nine (56%) recognized two of the antigens; and four (25%) recognized all three antigens. In terms of protein antigens, IE1 was recognized by 15 (94%) of the donors (predominantly by CD8^+^ T cells), pp65 was recognized by 13 (81%) of the donors (equally distributed between CD4^+^ and CD8^+^ T cell responses), and IE2 was recognized by five (31%) of the donors (predominantly by CD4^+^ T cells).

**Table 1:** Donor demographics.

	Ex Vivo T cell responses			Class I			Class II					
Donor#	pp65 CD4/8	IE1 CD4/8	IE2 CD4/8	HLA-A	HLA-B	HLA-C	DRB1	DRB3, 4, 5	DQA1	DQB1	DPA1	DPB1
1	+/+	−/+	−/−	02:01, 03:01	35:01, 44:02	04:01, 05:01	01:01, 11:01	3*02:02	01:01, 05:05	03:01, 05:01	01:03	04:01
5	+/+	−/+	−/+	01:01, 24:02	37:01, 39:06	06:02, 07:02	15:01	5*01:01	01:02	06:02	02:01	10:01, 11:01
8	+/+	−/+	−/−	11:01	55:01, 35:01	03:03, 04:01	01:01, 15:01	5*01:01	01:01, 01:02	05:01, 06:02	01:03	04:01, 04:02
13	−/+	−/+	−/−	02:01	39:01, 44:02	07:02, 07:04	07:01, 15:01	4*01:03, 5*01:01	01:02, 02:01	03:03, 06:02	01:03	04:01
14	−/−	−/+	+/−	01:01, 02:01	08:01, 37:01	06:02, 07:01	03:01, 09:01	3*01:01, 4*01:03	03:02, 05:01	02:01, 03:03	01:03	04:01
19	−/−	−/+	−/−	01:01, 02:01	08:01, 40:01	03:04, 07:01	03:01, 13:02	3*01:01, 3*03:01	01:02, 05:01	02:01, 06:04	01:XX	04:01
22	+/+	+/−	−/−	11:01, 32:06	13:02, 15:17	06:02, 07:01	07:01, 13:01	3*01:01, 4*01:03	01:03, 02:01	02:02, 06:03	01:03, 02:01	04:01, 17:01
23	+/+	−/+	+/−	01:01, 24:02	07:02, 08:01	07:01, 07:02	01:01, 03:01	3*01:01	01:01, 05:01	02:01, 05:01	01:03	04:01
26	+/−	−/−	−/−	01:01, 03:01	08:01, 27:05	02:02, 07:01	01:01, 04:01	4*01:03	01:01, 03:01	03:01, 05:01	01:03	04:01
28	+/−	+/+	−/−	03:01, 26:01	07:02, 14:01	07:02, 08:02	07:01, 08:03	4*01:01	02:01, 06:01	02:02, 03:01	01:03	02:01, 04:02
29	+/+	+/+	−/−	03:01, 11:01	13:02	06:02	07:01, 13:02	3*03:01, 4*01:03	01:02, 02:01	02:02, 06:04	01:03	04:01, 04:02
33	+/−	+/+	+/−	01:01	08:01	07:01	03:01	3*01:01	05:01	02:01	01:03	04:01
38	−/+	−/+	−/−	29:02, 68:01	44:02, 45:01	06:02, 07:04	07:01, 11:01	3*02:02, 4*01:01	02:01, 0505	02:02, 03:01	01:03	02:01, 04:02
40	−/+	−/+	−/−	01:01, 24:02	07:02, 39:06	07:02	01:01, 15:01	5*01:01	01:01, 01:02	05:01, 06:02	01:03	04:01, 04:02
41	+/−	+/−	+/−	01:01, 24:02	08:01, 38:01	07:01, 12:03	03:01, 07:01	3*01:01, 4*01:01	02:01, 05:01	02:01, 02:02	02:01	01:01, 11:01
44	−/−	−/+	−/−	01:01, 24:02	37:01, 40:01	03:04, 06:02	04:04, 08:01	4*01:03	03:01, 04:02	03:02, 04:02	01:03	02:01, 03:01

T cell responses were determined using *ex vivo* ICS. + denotes T cell response, - denotes no T cell response.

Sequence-based typing including all three loci encoding HLA class I molecules (HLA-A, -B, and -C) and all six loci encoding variable HLA class II molecules (HLA-DRB1, -DRB3/4/5, -DQA1, -DQB1, -DPA1 and -DPB1) were used to perform high-resolution HLA typing of the 16 donors (Table 1). These donors represent some of the most frequent HLA-types in the Caucasian population in Northern Europe ([48] and unpublished observations).

**Figure 1:**
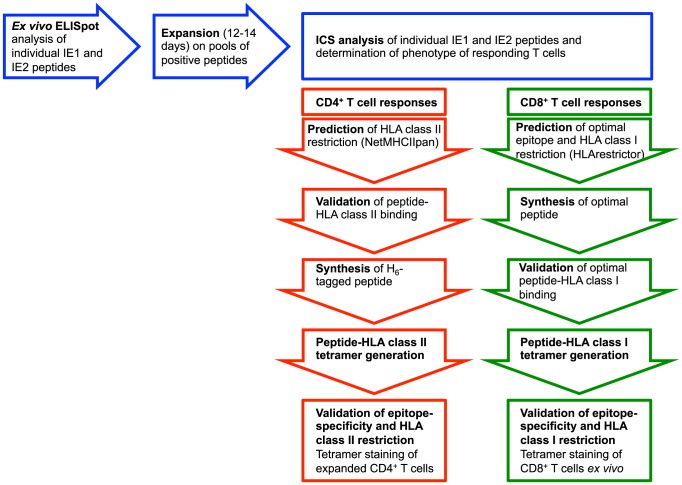
Overview of screening strategy. PBMCs were screened by *ex vivo* ELISpot analysis for recognition of 187 overlapping 15mer peptides spanning the entire IE1 and IE2. Pools of positively recognized peptides were used to expand the T cells for 12–14 days and subsequently analyzed for CD4^+^ and CD8^+^ T cell recognition using ICS and flow cytometric analysis. *CD4* ^+^ *T cell epitope deconvolution:* The recognized 15mer peptide and the donor's HLA class II molecules were submitted to NetMHCIIpan to predict the HLA class II restriction element and the peptide core sequence interacting with the HLA class II molecule. The interaction was subsequently validated using a biochemical HLA class II binding assay. For a selection of the recognized epitopes, H_6_-tagged peptides were produced and used to generate peptide-HLA class II tetramers, which were subsequently used for validation of T cell specificity and HLA class II restriction. *CD8^+^ T cell epitope deconvolution:* The 15mer peptides recognized by a given donor together with the donors HLA class I molecules were submitted to the HLArestrictor to predict the optimal size epitope and HLA-restriction. Interaction between the predicted epitope and HLA class I molecule was validated by biochemical affinity- and stability assays. The T cell specificity and HLA class I restriction was validated by peptide-HLA class I tetramer staining.

**Figure 2:**
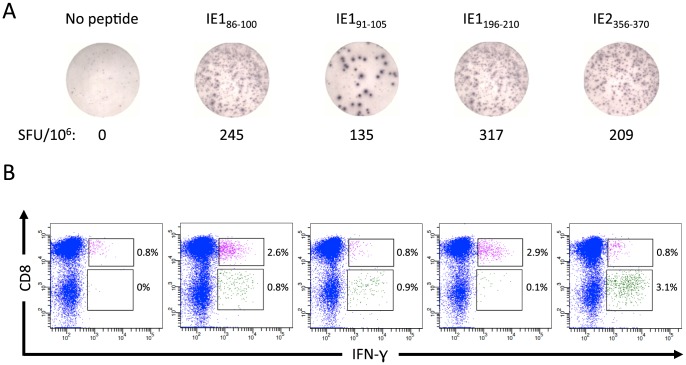
ELISpot and ICS analysis of donor 33. PBMCs were initially screened by *ex vivo* ELISpot and the CD4^+^ and CD8^+^ T cell recognition subsequently determined by ICS analysis. Donor 33 recognized four different peptides, which are indicated above the results. A) *Ex vivo* IFN-γ ELISpot assay. Spot forming units (SFU) are indicated as positive spots per 10^6^ PBMCs. B) ICS analysis of T cells expanded on the identified 15mers. FACS plots show gated CD3^+^ T cells. The elicited frequency is indicated. Pink indicates responding CD8^+^ T cells. Green indicates responding CD4^+^ T cells.

Having established that our cohort of donors all harbored HCMV-specific cellular immune responses we started screening for peptide-specific responses. We used two cellular assays, ELISpot and ICS, each with unique advantages and disadvantages. Whereas an ELISpot assay may be sensitive enough to capture *ex vivo* cellular responses, an ICS assay is less sensitive and may require *in vitro* culture and restimulation. On the other hand, an ICS assay can readily distinguish between CD4^+^ and CD8^+^ T cell responses. Using a multi-step procedure we combined the advantages of two assays: the ELISpot assay was initially used to identify individual *ex vivo* recognized peptides, next relevant peptides were used to expand the corresponding T cells *in vitro*, and then the ICS assay was used to characterize the expanded T cells identifying their peptide-specificity and CD4 or CD8 phenotype (this strategy is outlined in Figure 1, blue boxes). The combined screening procedure is illustrated here using donor 33, since this donor showed both CD4^+^ and CD8^+^ T cell reactivity, and had the added advantage of being homozygous, thus reducing the complexity of the HLA molecules involved in this example. According to the *ex vivo* ELISpot screening, donor 33 recognized four peptides: three IE1 peptides (IE1_86–100_, IE1_91–105_ and IE1_196–210_) and one IE2 peptide (IE2_356–370_) (Figure 2A). PBMCs were briefly *in vitro* cultured and expanded with a pool of these four peptides, and harvested. The cells were subsequently analyzed by ICS for CD4^+^ and CD8^+^ T cell recognition of the individual peptides: one peptide (IE1_196–210_) was only recognized by CD8^+^ T cells, another peptide (IE1_86–100_) was recognized by both CD4^+^ and CD8^+^ T cells, and two peptides (IE1_91–105_ and IE2_356–370_) were only recognized by CD4^+^ T cells (Figure 2B).

**Figure 3:**
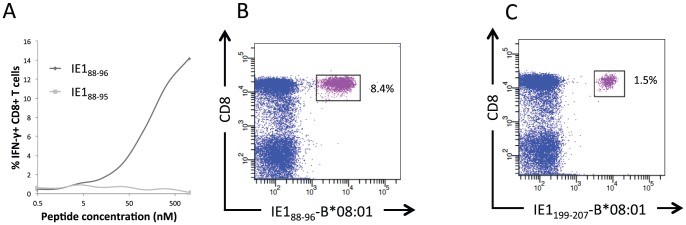
Donor 33 - CD8^+^ T cell epitope validation. CD8^+^ T cell responses were detected against the 15mers IE1_86–110_ and IE1_196–210_. In IE1_86–110_ two B*08:01 binding optimal peptides, IE1_88–95_ and IE1_88–96_, were predicted, while in IE1_196–210_ one B*08:01-restricted optimal peptide IE1_198–207_, was predicted. **A**) ICS analysis of predicted optimal peptides: Chart showing IFN-γ responses following restimulation of *in vitro* cultured PBMCs with graded doses of IE1_88–95_ and IE1_88–96_. **B**) Optimal epitope and HLA class I restriction validated by *ex vivo* peptide-HLA class I tetramer staining with the IE1_88–96_-HLA-B*08:01 tetramer. **C**) Optimal epitope and HLA class I restriction validated by *ex vivo* peptide-HLA class I tetramer staining with the IE1_199–207_-HLA-B*08:01 tetramer. The plots show gated CD3^+^ T cells; frequencies of tetramer-positive CD8^+^ T cells (boxed-in and pink) are indicated.

The ideal way to identify and validate the epitope-specific, HLA-restricted specificity of a T cell response, would be to label the T cells with specific HLA tetramers; peptide-HLA class I tetramers for CD8^+^ T cell responses, and peptide-HLA class II tetramers for CD4^+^ T cell responses (the strategies for the generation of appropriate HLA class I and II tetramers are outlined in Figure 1, green and red boxes, respectively). To generate appropriate peptide-HLA class I tetramers one would have to identify the proper peptide-MHC combination recognized by the T cell in question. A priori, any submer peptide (e.g. 8-, 9-, 10-, and 11mers), that potentially could be generated from a given 15mer peptide and bind to any of the donor's HLA class I molecules, could be involved. Our recently described bioinformatics predictor, HLArestrictor [42], aims at simplifying this process by predicting which combinations of submer peptide and available HLA class I molecule are the most likely T cell receptor ligands. In this case, CD8^+^ T cells from donor 33 with HLA-A*01:01, -B*08:01, and -C*07:01 recognized the two 15mer peptides IE1_86–100_ and IE1_196–210_. The highest-ranking peptide-HLA combinations predicted for the 15mer peptide IE1_196–210_ was the 9mer IE1_199–207_-HLA-B*08:01, with a percentile rank of the predicted affinity of 0.1 (i.e. less than 1 out of 1000 random peptides are predicted to bind with better affinity). Among the highest-ranking peptide-HLA combinations predicted for the 15mer peptide IE1_86–100_ was the 8mer IE1_88–95_-HLA-B*08:01 and 9mer IE1_88–96_-HLA-B*08:01 with percentile ranks of predicted binding affinities of 1.5 and 3.0, respectively. These three submer peptides were synthesized and their binding status were validated in a biochemical peptide-HLA class I binding assay (Table 2). Conventionally, a threshold of 500 nM affinity is expected for MHC class I-restricted epitopes (however, we have noted that the peptide-binding affinities to our recombinant HLA-B*08:01 is lower than for most other HLA class I molecules, and we are currently trying to understand whether this is an artifact of the recombinant molecules, or a real phenomenon). Although low, these binding affinities were sufficient to support folding and tetramer production for the IE1_88–96_-HLA-B*08:01 combination. *In vitro* restimulated CD8^+^ T cells from donor 33 responded to IE1_88–96_, but not to IE1_88–95_ (Figure 3A). Finally, the epitope and restriction specificities could be validated by *ex vivo* tetramer staining of PBMCs from donor 33. Both IE1_88–96_-HLA-B*08:01 and IE1_198–207_-HLA-B*08:01 tetramers labeled a high frequency of the CD8^+^ T cells: 8.4% were labeled by IE1_88–96_-HLA-B*08:01 and 1.5% by IE1_198–207_-HLA-B*08:01 (Table 2; Figure 3B and C). Thus, the specificities of both CD8^+^ T cell responses identified in donor 33 had been defined and validated. Each peptide represented a CD8^+^ T cell epitope restricted by HLA-B*08:01.

**Table 2:** Donor 33 CD8^+^ T cell responses.

15-mer peptide	Sequence	SFU/10^6^ PBMC	ICS (%)	Submer peptide	Sequence	ICS (%)	HLA-	Predicted affinity (RANK)	Measured affinity (nM)	Stability (t½; h)	TMRI (%)
IE1_86–100_	VKQIKVRVDMVRHRI	245	2.6	IE1_88–95_	QIKVRVDM	0	B*08:01	0.015	579	0.5	NA
				IE1_88–96_	QIKVRVDMV	9.9	B*08:01	0.03	994	0.4	8.4
IE1_196–210_	KKDELRRKMMYMCYR	209	2.1	IE1_199–207_	ELRRKMMYM	4.9	B*08:01	0.001	105	0.6	1.5

ELISpot results, ICS results following *in vitro* culture, predicted affinity, measured peptide-HLA class I binding (affinity and stability), and *ex vivo* HLA class I tetramer staining results (TMRI) are shown. SFU: Spot forming units indicated as spots per 10^6^ PBMCs.%: percentage of CD8^+^ T cells stained. ND: not done. NA: Not applicable

To generate appropriate peptide-HLA class II tetramers one would have to identify which of the HLA class II molecules available to the donor could serve as restriction element(s). Whereas the design of HLA class I tetramers also included an identification of the optimal peptide, a similar step was not needed for the design of HLA class II tetramers since longer peptides can extend out of the HLA class II molecule at either end of the peptide-binding cleft. Our prediction tool NetMHCIIpan [43] was used to predict the most likely HLA class II restriction element(s) for the identified CD4^+^ T cell epitopes. In addition, the identified peptides were evaluated for binding to the available HLA class II molecules of the donor in a biochemical peptide-HLA class II binding assay. At this point the technologies for HLA class II production, peptide binding analysis and predictions are more mature for the HLA-DR molecules, than for HLA-DQ and -DP molecules. In addition, we have recently developed an HLA class II tetramer protocol employing H_6_-tagged peptides for purification purposes, which has been validated for several HLA-DR molecules[41]. Thus, out of necessity and practicality, we have focused our HLA class II restriction analysis on HLA-DR molecules.

**Figure 4:**
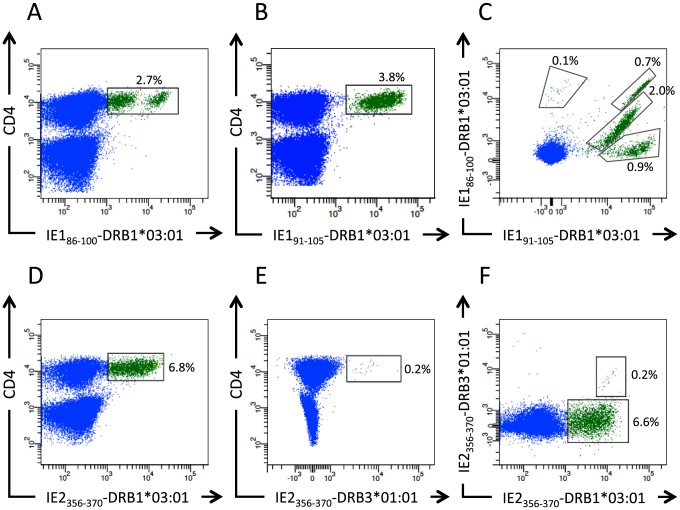
Donor 33 - CD4^+^ T cell epitope validation. Three different 15mers elicited CD4^+^ T cell responses, IE1_86–100_, IE1_91–105_, and IE2_356–370_ in donor 33. T cells were expanded for 12 days on a mix of the three peptides. The specificity and HLA class II restriction of the CD4^+^ T cell responses were evaluated using peptide-HLA class II tetramers. The cells were stained with anti-CD3, -CD4, and the peptide-HLA class II tetramers either alone or in combination. IE1_86–100_ and IE1_91–105_ are overlapping peptides and bind to the same DRB1*03:01. The cells are stained in A) with the IE1_86–100_-DRB1*03:01 tetramer, in B) with the IE1_91–105_-DRB1*03:01 tetramer, and in C) with a combination of PE-labeled IE1_86–100_-DRB1*03:01 and APC-labeled IE1_91–105_-DRB1*03:01 tetramer. The IE2_356–370_ peptide binds to both of the donor's HLA-DR molecules, DRB1*03:01 and DRB3*01:01. The cells are stained in D) with the IE2_356–370_-DRB1*03:01 tetramer, in E) the IE2_356–370_-DRB3*01:01 tetramer, and in F) with a combination of PE-labeled IE2_356–370_-DRB1*03:01 and APC-labeled IE2_356–370_-DRB3*01:01 tetramer. The plots show gated CD3^+^ T cells, and the frequency of tetramer-positive CD4^+^ T cells (boxed-in and green) is indicated.

Donor 33 had CD4^+^ T cell responses directed against three peptides: two overlapping peptides, IE1_86–100_ and IE1_91–105_, and a singular peptide, IE2_356–370_. These were evaluated for binding to the two HLA-DR molecules of this donor (Table 3). The two overlapping peptides IE1_86–100_ and IE1_91–105_ were both predicted and measured to be high affinity binders to HLA-DRB1*03:01 (measured binding affinities of 4 nM and 10 nM, respectively), and both supported tetramer generation. Since CD4^+^ T cells tend to be present at low frequencies[49], PBMCs were *in vitro* expanded before labeling them with HLA class II tetramers. Both overlapping tetramers labeled expanded CD4^+^ T cells: IE1_86–100_-HLA-DRB1*03:01 tetramers labeled 2.7% and IE1_91–105_-DRB1*03:01 tetramers labeled 3.8% of the CD4^+^ T cells (Figure 4A and B). One of the overlapping peptides, IE1_86–100_, also bound to HLA-DRB3*01:01 and supported tetramer generation, but no IE1_86–100_-HLA-DRB3*01:01 tetramer labeling of CD4^+^ T cells could be detected (Table 3). In addition to predicting the binding affinity between a given peptide and an HLA class II molecule, NetMHCIIpan also predicts the core sequence of the peptide interacting with the HLA class II molecule. Thus, peptides IE1_86–100_ and IE1_91–105_ were predicted to bind to HLA-DRB1*03:01 through the same core sequence (VRVDMVRHR). This raises the possibility that it might be the same, or largely overlapping, T cell populations that recognize the two complexes. To determine this, the cells were double labeled with PE-labeled IE1_86–100_-HLA-DRB1*03:01 and APC-labeled IE1_91–105_-HLA-DRB1*03:01 (Figure 4C). Labeling with the IE1_86–100_-HLA-DRB1*03:01 tetramer indicates that there are at least two CD4^+^ T cell populations recognizing the epitope with different affinities (Figure 4A). The double labeling splits this up even further since there are at least four different CD4^+^ T cell populations recognizing the two epitopes with different affinities as judged by their staining intensities: One population (0.9%) recognizing only the IE1_91–105_-DRB1*03:01 epitope, two populations accounting for the majority of the CD4^+^ T cells (2% and 0.7%), which recognize both epitopes with different affinities, and one very small population (0.1%) recognizing only the IE1_86–100_-DRB1*03:01 epitope (Figure 4C). During processing of an epitope various sizes might be produced that all bind through the same core sequence with various flanking sequences that can stimulate different T cell clones thereby broadening the T cell repertoire, which in principal could recognize the same epitope[50].

**Table 3:** Donor 33 CD4^+^ T cell responses.

				DRB1*03:01				DRB3*01:01			
Peptide	Sequence	SFU/10^6^ PBMC	ICS (%)	Predicted core sequence	Predicted affinity (nM)	Measured affinity (nM)	TMRII (%)	Predicted core sequence	Predicted affinity (nM)	Measured affinity (nM)	TMRII (%)
IE1_86–100_	VKQIKVRVDMVRHRI	245*	0.8	VRVDMVRHR	5	4	2.7	VRVDMVRHR	63	109	0
IE1_91–105_	VRVDMVRHRIKEHML	135	0.9	VRVDMVRHR	50	10	3.8	VRVDMVRHR	1215	>5000	NA
IE2_356–370_	TRRGRVKIDEVSRMF	209	3.1	VKIDEVSRM	12	9	6.8	VKIDEVSRM	52	2	0.2

ELISpot results, ICS results following *in vitro* culture, predicted core and affinity, measured peptide-HLA class II affinity, and *in vitro* HLA class II tetramer staining results are shown (TMRII). SFU: Spot forming units indicated as spots per 10^6^ PBMCs. NA: Not applicable. *: Response includes a CD8^+^ T cell epitope

The third peptide, IE2_356–370_, was a high affinity binder (K_D_<50 nM) to both DRB1*03:01 and DRB3*01:01 (Table 3). Subsequent tetramer labeling showed that the CD4^+^ T cells predominantly recognized IE2_356–370_ presented by HLA-DRB1*03:01 (labeling 6.8% of the expanded CD4^+^ T cells) and to a lesser extent IE2_356–370_ presented by HLA-DRB3*01:01 (0.2%) (Figure 4D and E). Tetramer double staining analysis revealed that the latter tetramer labeling could be fully accounted for by a small CD4^+^ T cell population that cross-recognized the epitope presented by both DRB1*03:01 and DRB3*01:01 (Figure 4F). Note, that the two HLA-DR molecules were predicted to bind the same core sequence of the peptide, which could explain how a small CD4^+^ T cell population could recognize the same peptide in the context of two different HLA-DR molecules (Table 3).

Thus, the specificities of all three CD4^+^ T cell responses identified in donor 33 had been defined and validated. The overlapping peptides IE1_86–100_ and IE1_91–105_ represented one epitope presented by one HLA-DR molecule, HLA-DRB1*03:01. The remaining peptide, IE2_356–370_, represented one epitope presented by two HLA-DR molecules; primarily by HLA-DRB1*03:01 and to lesser extent by HLA-DRB3*01:01. Note that no attempts to map these CD4^+^ responses to HLA-DQ or -DP have been made, and that we cannot exclude the existence of additional CD4^+^ T cell responses restricted to these HLA class II isotypes.

### Extending the identification of CD8^+^ T cell epitopes to all donors

The study was subsequently extended to 15 additional donors, who were screened for recognition of the 187 overlapping IE1 and IE2 peptides. The initial ELISpot screening revealed that each donor recognized from zero to 15 of the overlapping IE1 and IE2 peptides (data not shown)). The subsequent ICS analysis of *in vitro* stimulated and cultured T cells showed that per donor zero to six 15mer peptides elicited CD8^+^ T cell responses (one donor, donor 26, did not have any detectable anti-IE1 or -IE2 CD8^+^ T cell responses), whereas two to ten 15mer peptides elicited CD4^+^ T cell responses (Table 4). Three donors recognized CD8^+^ T cell epitopes located within the initial 85 amino acid sequence, which is identical in IE1 and IE2, but somewhat surprisingly, none of the donors recognized CD8^+^ T cell epitopes located within the unique part of IE2 (Table 4).

**Table 4:** IE1 and IE2 T cell responses identified per donor.

	IE1/IE2 shared segment		Unique IE1 segment		Unique IE2 segment		IE total	
Donor#	CD4	CD8	CD4	CD8	CD4	CD8	CD4	CD8
1	0	0	0	3	4	0	4	3
5	0	0	4	3	2	0	6	3
8	0	0	1	1	3	0	4	1
13	0	0	1	1	1	0	2	1
14	0	0	3	3	5	0	8	3
19	0	2	2	4	1	0	3	6
22	0	0	6	3	1	0	7	3
23	0	0	5	1	5	0	10	1
26	0	0	0	0	2	0	2	0
28	0	0	2	2	2	0	4	2
29	0	0	4	5	4	0	8	5
33	0	0	2	2	1	0	3	2
38	0	1	2	2	3	0	5	3
40	0	0	1	1	3	0	4	1
41	0	0	7	4	3	0	10	4
44	0	2	1	4	0	0	1	6
**Total**	0	5	41	39	40	0	81	44

Sixteen donors were evaluated for CD4^+^ and CD8^+^ T cell recognition of overlapping IE1 and IE2 peptides. The initial segment of IE1 and IE2 (85 amino acids) is identical and thus the proteins have been divided in three segments: shared IE1 and IE2, unique IE1, and unique IE2. T cell responses are shown in terms of ICS validated responses against individual 15mer peptides

In the 16 donors, a total of 44 CD8^+^ T cell responses were observed against 16 different HCMV-derived IE1 and/or IE2 peptides. For each peptide, the peptide sequence and the HLA class I molecules of each responding donor were submitted to the HLArestrictor, which then suggested the most likely peptide-HLA class I combinations. Shared HLA class I molecules between the responding donors were taken into account when selecting which predicted epitopes should be synthesized. Once these peptides had been acquired, they were analyzed for binding to the suggested HLA class I restriction elements, and for the stability of the resulting peptide-HLA class I complexes (stability has been suggested to be a better correlate of immunogenicity than affinity [51]). For all productive interactions, peptide-HLA class I tetramers were generated and used to label relevant CD8^+^ T cells. The 16 overlapping peptides gave rise to 14 different tetramer validated CD8^+^ T cell epitopes covering 11 different HLA class I restriction elements (three of the epitopes were found in overlapping peptides and one 15mer peptide contained two epitopes, an 8mer and 9mer, restricted by two different HLA class I molecules, B*08:01 and C*06:02, thus accounting for all 16 overlapping peptides) (Table 5; several epitopes have been described elsewhere[52], [53], [54], [55], [56], [57], [58], [59], [60], [61]). Twelve of the 14 validated CD8 epitopes represented interactions with a half-life longer than 1 h (Table 5) emphasizing the importance of peptide-HLA class I stability in defining immunogenicity. In total, 44 of 44 (100%) CD8^+^ T cell responses that had been detected by the combined ELISpot/ICS approach were explained and validated at the level of tetramer labeling.

**Table 5:** Summary of identified CD8^+^ T cell epitopes.

Peptide	Sequence	HLA restriction	Predicted affinity (%RANK^a^)	Measured affinity (nM)	Stability (T_½_; h)	TMRI (%)	Responders/donors tested	Responding donors	Reference
IE1_33–41_/IE2_33–41_	TTFLQTMLR	A*68:01	0.03	1	57.4	0.1	1/1	38	[52] b, this paper
IE1_42–50_/IE2_42–50_	KEVNSQLSL	B*40:01	0.10	25	1.2	0.1–0.3	2/2	19, 44	[53]
IE1_88–95_	QIKVRVDM	C*06:02	NB	3	8.8	0.1–0.5	4/6	22, 29, 38, 44	[56] c, this paper
IE1_88–96_	QIKVRVDMV	B*08:01	3.00	994	0.4	0.1–14	4/6	14, 19, 33, 41	[54]
IE1_99–107_	RIKEHMLKK	A*03:01	0.10	542	33.6	0.1–0.4	2/4	1, 29	[61]
IE1_184–192_	KLGGALQAK	A*03:01	0.80	216	46.1	0.1–6.2	3/4	1, 28, 29	this paper
IE1_199–207_	ELRRKMMYM	B*08:01	0.10	105	0.6	0.1–4.4	4/6	14, 19, 33, 41	[54]
IE1_221–231_	FPKTTNGCSQA	B*55:01	0.25	138	6.0	10.3	1/1	8	[52], [55]
IE1_248-256_	AYAQKIFKI	A*24:02	0.25	9	35.6	0.1–3.1	3/5	5, 41, 44	[57]
IE1_290–299_	TSDACMMTMY	A*01:01	0.01	9	8.5	0.1–1.2	4/9	5, 14, 41, 44	[57] d, this paper
IE1_309–317_	CRVLCCYVL	C*07:02^f^	NB	ND	12.6	0.8–8.0	5/5	5, 13, 23, 28, 40	[58] e, this paper
IE1_316–324_	VLEETSVML	A*02:01	4.00	23	1.8	0.4	1/4	1	[59]
IE1_354-363_	YILGADPLRV	B*13:02	NB	87	4.4	0.1–0.2	2/2	22, 29	[60] g, this paper
IE1_381–389_	EEAIVAYTL	B*40:01	0.17	13	1.3	7.3	1/2	19	[52] h, this paper

a :%RANK: The percentage of random peptides with a predicted affinity stronger than the candidate epitope.

b : Previously reported as an N-terminally extended 10mer.

c : Previously reported as an N-terminally extended 9mer.

d : Previously reported as an N-terminally truncated 9mer.

e : Previously reported as B*07:02-restricted.

f : Also determined by mixed allo presentation.

g : Previously reported as A*02:01-restricted.

h : Previously reported as B*44:02-restricted. NB: Non-binder; predicted affinity is usually weaker than the predicted affinity of the upper 2% of random peptides. ND: not done. TMRI: *Ex vivo* HLA class I tetramer staining results.

### Extending the identification of CD4^+^ T cell epitopes to all donors

In the 16 donors, a total of 81 CD4^+^ T cell responses were observed against 28 different HCMV peptides derived from IE1 and/or IE2; 14 of the 28 peptides were derived from IE1 (some have previously been described[41], [62], [63], [64]) and 14 from IE2, and all donors responded with an almost equal distribution of IE1 and IE2 epitopes (Table 4). Many of the peptides (16 of 28) were recognized in two or more of the 16 donors. To evaluate the possible HLA class II restriction elements we focused on the HLA-DR isotype due to the availability of predictions and measurements of peptide-binding, and the possibility of generating HLA class II tetramers. This is also appropriate from a functional perspective since 89% of reported HLA class II restrictions have been HLA-DR-restricted (IEDB, August 2013). Thus, all peptide-HLA-DR combinations were submitted to the NetMHCIIpan predictor to identify the most likely HLA-DR restriction element and to identify the core sequence involved in HLA-DR binding; and peptide-HLA-DR binding was measured whenever possible (the 16 donors had 18 different HLA-DR molecules in total; 14 of these were available for binding analysis). The predicted and/or measured binding between the recognized peptide and the HLA-DR molecules are shown in Tables S1 and S2. In accordance with the general assumption that HLA class II molecules are more promiscuous than HLA class I molecules [65], several peptides bound strongly to multiple HLA-DR-molecules or were predicted to do so. For each peptide in these tables, we have underlined the most likely peptide-HLA class II combination(s) based on high binding affinity (we have arbitrarily chosen a binding cut-off of 500 nM) and whether the HLA class II molecule was shared between several responding donors.

In several cases, the above analysis still left multiple HLA class II molecules as being possible restriction elements. An ideal way to resolve the specificity of CD4^+^ T cells is to use HLA class II tetramers, however, the availability of HLA class II tetramers is quite limited. We have recently developed a “tagged peptide” approach to HLA class II tetramer generation. Guided by peptide-HLA-DR affinity and by shared HLA-DR molecules within the responding donors, we generated peptide-HLA DR tetramers for a limited number of the most frequently recognized epitopes. Thus, 10 peptides were selected and produced as H_6_-tagged peptides for HLA-DR tetramer production. Using these tetramers we successfully validated the HLA class II restriction for eight of the 28 15mer peptides that elicited CD4^+^ T cell responses (also included in Tables S1 and S2, and summarized in Table 6). This limited panel of peptide-HLA-DR combinations included at least seven epitopes (using core sequences as an approximation, the following epitopes were identified: VRVDMVRHR, IKEHMLKKY, FTKNSAFPK, VKIDEVSRM, QIIYTRNHE, IIYTRNHEV, and FLMEHTMPV) and seven different HLA-DR molecules (HLA-DRB1*01:01, -DRB1*03:01, -DRB1*07:01, -DRB1*13:01, -DRB1*15:01, DRB3*01:01, and -DRB5*01:01) allowing tetramer validation of 39 of the 81 observed CD4^+^ responses. In the future, we expect more CD4^+^ T cell responses to be tetramer validated as HLA class II tetramers become more widely available.

**Table 6:** Summary of HLA class II tetramer validated IE1/IE2 epitopes.

				Validated HLA class II restriction			
Peptide	Sequence	Donors	Total # of donors	Restriction	Core sequence predicted	measured affinity (nM)	Reference
IE1_86–100_	VKQIKVRVDMVRHRI	14, 19, 23, 33, 41	5	DRB1*03:01	VRVDMVRHR	4	this paper
IE1_91–105_	VRVDMVRHRIKEHML	14, 19, 23, 33, 41	5	DRB1*03:01	VRVDMVRHR	10	[63], [64]
		22	1	DRB1*13:01	VRVDMVRHR	2	
IE1_96–110_	VRHRIKEHMLKKYTQ	22	1	DRB1*13:01	IKEHMLKKY	2	[63], [64]
IE1_211–225_	NIEFFTKNSAFPKTT	5, 8, 13, 40	4	DRB5*01:01	FTKNSAFPK	7	[41]
IE2_356–370_	TRRGRVKIDEVSRMF	14, 19, 23, 33, 41	5	DRB1*03:01	VKIDEVSRM	9	this paper
		33^a^	1	DRB3*01:01	VKIDEVSRM	2	
IE2_408–422_	KGIQIIYTRNHEVKS	5, 13^b^, 40	3	DRB1*07:01	IIYTRNHEV	53	this paper
		13^b^, 22, 28, 29, 38, 41	6	DRB1*15:01	QIIYTRNHE	45	
IE2_438–452_	ALSTPFLMEHTMPVT	1, 8, 23, 26, 40	5	DRB1*01:01	FLMEHTMPV	6	this paper
IE2_443–457_	FLMEHTMPVTHPPEV	1, 8, 23, 26, 40	5	DRB1*01:01	FLMEHTMPV	2	this paper

a : A subpopulation of T cells could also be stained with an IE2_356–370_-HLA-DRB3*01:01 tetramer. This phenomenon was not observed in donor 14, 19, or 23 (donor 41 not done).

b : T cell populations that could be labeled with IE2_408–422_-DRB1*07:01 or IE2_408–422_-DRB1*15:01 were detected.

## Discussion

The IE1 and IE2 proteins are among the first to be expressed during HCMV infection and reactivation. These proteins may therefore be particularly valuable targets for an immune based prevention and/or treatment strategy against HCMV. A priori, an approach exploiting multiple epitopes, multiple HLA restriction elements, and encompassing both CD8^+^ and CD4^+^ T cell responses, should stand a better chance of generating a robust, long-lived response and avoid virus escape. Here, we have used 187 overlapping peptides representing the complete 412 and 580 amino acid long IE1 and IE2 protein sequences, respectively, to investigate the spectrum of IE1- and IE2-specific T cell responses in human donors. Examining 16 HCMV-reactive donors, we identified a total of 44 CD8^+^ T cell responses involving 16 peptides, and 81 CD4^+^ T cell responses involving 28 overlapping peptides (corresponding to about three CD8^+^ and 5 CD4^+^ T cell responses per donor per 1000 amino acids). These peptides were then analyzed by peptide-HLA class I or II binding predictions and/or measurements suggesting suitable peptide-HLA combinations for subsequent investigations. Whenever possible, relevant peptide-HLA tetramers were generated and used to examine and validate the peptide-specific, HLA-restricted nature of the observed T cell reactivities.

All CD8^+^ T cell epitopes found in the initial screen could eventually be identified and validated at the tetramer level. Several factors contributed to this success rate. With current technology, it was feasible to acquire a systematic set of overlapping peptides representing the entire IE1 and IE2 proteins. It was also feasible to obtain sufficient numbers of donor T cells to test T cell responses against these proteins. This allowed us to use functional T cell assays such as ELISpot and/or ICS as the initial epitope screen. Only then were biochemical (in particular peptide-HLA class I stability measurements) and bioinformatics approaches used to search for T cell epitopes within the overlapping 15mer peptides that gave a positive hit in the initial screen, and to search for their HLA restriction elements. This strategy of using a functional screen first and a bioinformatics screen second has the advantage that it avoids many of the false positives that plague peptide-HLA predictors when they are used as the initial screen. Under these conditions, the HLArestrictor proved to be a very efficient tool to identify CD8^+^ T cell epitopes and their HLA restriction elements. In about 90% of the T cell responses found in the initial screen, the HLArestrictor successfully identified the peptide-HLA combination that later could be validated by HLA tetramer analysis; in fact, in 65% it was the very first choice of the HLArestrictor. The output from the HLArestrictor was used to select new peptides for synthesis as putative CD8^+^ T cell epitopes and then to generate the corresponding peptide-HLA class I tetramers, which were used to validate the proper CD8^+^ T cell epitopes. Here, we successfully used our “one-pot, mix-and-read” HLA class I tetramer technology[40] to generate 14 HLA class I tetramers covering 11 different HLA class I molecules. Thus, we have established a highly efficient mode of CD8^+^ T cell discovery suitable for smaller target antigens. This approach would even be suitable for a small virus proteome of a few thousand amino acids; however, with current technologies both the costs of peptides and the limited size of donor samples prohibit the application of this approach to T cell epitope discovery involving larger challenges (e.g. above large viral proteomes). Extending this highly efficient approach to larger challenges would require that future technologies manage to miniaturize the initial functional screen e.g. through peptide or peptide-HLA microarrays[66], [67], [68], [69], [70]. One potential drawback of our strategy is that we might miss low frequency epitopes that are not detectable by the *ex vivo* ELISpot analysis as well as the occasional CD8 epitopes that cannot be efficiently generated by processing during the *in vitro* culture of the 15mer peptide[71]. Some of the results gave rise to redefinitions of previously published epitopes in terms of peptide-length and/or HLA restriction (see File S1).

In contrast to the high success rate of our CD8^+^ T cell discovery strategy, only about half of the CD4^+^ T cell responses could be explained and validated at the level of peptide-HLA class II tetramers (Tables S1 and S2). This is in line with the notion that HLA class I technologies currently are more mature with more accurate predictions[72], [73], having better coverage with respect to predicting and measuring peptide-binding to HLA class I, and better availability of HLA class I tetramers than the corresponding HLA class II technologies[40], [42], [43], [44], [46], [74], [75]. Thus, the exact identification of the specificity of a CD4^+^ T cell response can be quite cumbersome. In particular, it is not trivial to establish which HLA class II molecule is involved as restriction element in a given CD4^+^ T cell response. This is often inferred based on more or less indirect assays such as predictions or measurements of peptide-HLA class II interactions, the inhibition mediated by antibodies specific for known HLA iso- or allo-types, presentation by HLA matched or unmatched cell lines etc. The latter is indeed the path taken by Sette and coworkers, who recognized that the problems of establishing HLA class II restrictions are so manifest, that they recently established a panel of 46 single-transfected cell lines with the primary purpose of allowing accurate determination of HLA class II restriction[76]. The interpretation of these indirect assays is complicated by the promiscuous nature of peptide binding to HLA class II and of T cell recognition as exemplified here. Thus, two epitopes were presented in the context of more than one restriction element (e.g. IE1_91–105_ with DRB1*03:01 and DRB1*13:01; IE2_408–422_ with DRB1*07:01 and DRB1*15:01); and different extensions of the same core-peptide presented by the same HLA class II molecule, as well as the same peptide presented by different HLA class II molecules, were seen by some T cells as being identical, yet by other T cells as being dissimilar (see Figure 4C and 4F). Ideally, one would like to use peptide-HLA class II tetramers to identify and validate CD4^+^ T cell epitopes. Here, we have used our recently reported HLA class II tetramer technology[41] to generate peptide-HLA class II tetramers. This allowed us to validate about half of the observed CD4^+^ T cell responses (Table 6). At this time, our availability of HLA class II tetramers is limited to HLA-DR molecules. For 25 of the 28 peptides that stimulated CD4^+^ T cell responses we were able to suggest an HLA-DR restriction element. No HLA-DR binding could be detected for the remaining three peptides (IE1_429–448_, IE1_449–463_ and IE1_453–468_). These cases could represent CD4^+^ T cell responses that are restricted to HLA-DQ or -DP molecules. These examples emphasize the need for extending efficient bioinformatics, immunochemistry, and tetramer technologies to these isotypes, too.

The phenomenon that two overlapping peptides both stimulated a CD4^+^ T cell response in the same donor was a very frequent observation (IE1_81–95_ /IE1_86–100_; IE1_86–100_/IE1_91–105_; IE1_91–105_/IE1_96–110_; IE1_449–463_/IE1_453–468_; IE2_151–165_/IE2_156–170_; IE2_383–397_/IE2_388–402_; IE2_438–452_/IE2_443–457_; and IE2_558–572_/IE2_563–577_). As exemplified here, these overlapping pairs may represent one and the same epitope being present in both overlapping peptides. Alternatively, they may represent two different epitopes. As illustrated here double labeling using tetramers corresponding to each of the overlapping peptides should be able to validate whether overlapping responses represented a single unifying specificity, or two distinct specificities. Ideally, such cross-reactions should be captured and indicated by the core sequences suggested by the NetMHCIIpan predictor. Indeed, the same core-sequences were suggested for IE1_86–100_ and IE1_91–105_ binding to HLA-DRB1*03:01 (Table 6 and Table S1), which were cross-recognized (Figure 4C) as well as for the IE2_356–370_ peptide binding to both HLA-DRB1*03:01 and HLA-DRB3*01:01 (Table 6 and Table S2), which were also cross-recognized (Figure 4F).

Some of the HLA class II epitopes were recognized very frequently in our donors. A single epitope, IE2_408–422_, was recognized in 10 of the 16 donors (Table S2). Three donors, who expressed HLA-DRB1*15:01, all had CD4^+^ T cells with tetramer validated specificity for IE2_408–422_ presented by HLA-DRB1*15:01, and six donors, who expressed HLA-DRB1*07:01, had CD4^+^ T cells with tetramer validated specificity for IE2_408–422_ presented by HLA-DRB1*07:01. Of note, one donor had CD4^+^ T cells recognizing IE2_408–422_ presented by both HLA-DRB1*07:01 and HLA-DRB1*15:01, and in two donors we were not able to determine the restriction element(s). Tetramers with the IE2_408–422_ epitope were also synthesized with HLA-DRB1*01:01, HLA-DRB1*03:01, HLA-DRB1*11:01, HLA-DRB1*13:01, HLA-DRB4*03:01, and HLA-DRB5*01:01, but these did not label CD4^+^ T cells in relevant donors. A cluster of four overlapping IE1 peptides covering amino acid 81 to 110 in IE1 were recognized in seven donors. These epitopes appeared in pairs of two overlapping peptides (i.e. the overlapping IE1_81–95_ and IE1_86–100_ epitopes were recognized by two donors, the overlapping IE1_86–100_ and IE1_91–105_ epitopes were recognized by five donors, and the overlapping IE1_91–105_ and IE1_96–110_ epitopes were recognized by one donor) (Table S2). The two donors, who recognized the overlapping IE1_81–95_ and IE1_86–100_ peptides, shared HLA-DRB4*01:03, -DRB1*13:01 and -DRB1*13:02, all of which, from a peptide binding point of view, could serve as restriction elements. The five donors, who recognized the overlapping IE1_86–100_ and IE1_91–105_ peptides, shared HLA-DRB1*03:01, which bound both peptides with high affinity. PBMCs from all five donors could be labeled with HLA class II tetramers generated with HLA-DRB1*03:01 and peptides IE1_86–100_ or IE1_91–105_. Labeling the PBMCs with both tetramers revealed that we are dealing with a dominant TcR specificity, which reacts with both tetramers, and minor populations, which react with only one of the two tetramers (see Figure 4C for donor 33, who was one of these five donors). In agreement with the dominant shared specificity, the NetMHCIIpan predictor identified the same core sequence from the two overlapping peptides. The one donor, who recognized the overlapping IE1_91–105_ and IE1_96–110_ peptides, expressed one HLA class II molecule that was predicted and measured to be a binder of both peptides: HLA-DRB1*13:01. The two corresponding tetramers, IE1_91–105_ -DRB1*13:01 and IE1_96–110_-DRB1*13:01, were generated and the majority of the CD4^+^ T cells appeared to react with both tetramers. In this case, however, the NetMHCIIpan predictor suggested different core sequences from the two overlapping peptides.

In a landmark study by Sylwester and coworkers, who analyzed the immunogenicity of 213 HCMV open reading frames, IE1 and IE2 were among the most immunogenic proteins, inducing CD4^+^ and or CD8^+^ T cell responses in a high percentage of healthy seropositive donors. Furthermore, the average response frequencies of memory CD4^+^ and CD8^+^ T cells against the two proteins were comparable although CD4^+^ T cell response magnitudes directed against IE1 and IE2 were not as strong as CD4^+^ T cell responses directed against pp65[15]. Other studies [53], [56] have not been able to reproduce the CD4^+^ T cell immunogenicity against IE1 observed by Sylwester and coworkers. Our findings are more consistent with later reports as detection of CD4^+^ T cell responses against IE1 relied on ELISpot and subsequent *in vitro* stimulation. This applies to IE2 as well, but here the absence of apparent *ex vivo* immunogenicity was also observed in the CD8^+^ T cell population. Numerous T cell epitopes have been identified in IE1, most being CD8^+^ T cell epitopes, but also CD4^+^ T cell epitopes[41], [52], [53], [54], [55], [56], [58], [59], [60], [61], [62], [63], [64]. To our knowledge less than a handful IE2 epitopes have been published[77], [78], [79]. Here, we have found several novel and immunodominant CD4^+^ and CD8^+^ T cell epitopes. In IE1, we found both CD4 and CD8 epitopes, whereas CD4^+^ T cell epitopes dominated in IE2. In general, each individual donor recognized more CD4 epitopes than CD8 epitopes, but the frequencies of CD8^+^ T cells were higher than the frequencies of CD4^+^ T cells. Involving merely 16 donors, this study has generated information about CD4^+^ and CD8^+^ T cell epitopes against IE1/IE2 that potentially cover 91 and 98% of the Caucasian population, respectively (ignoring linkage disequilibrium). We believe that IE1 and IE2 represent a source of particularly important HCMV-derived immune targets, and that the information obtained here should provide important information for future vaccine development and adoptive T cell transfer against HCMV. Due to the detrimental effects of HCMV during fetal development and in immunocompromised patients, generating efficient vaccines and/or immunotherapies against HCMV remain a very high priority. We are currently addressing the importance of these epitopes, as well as pp65 T cell epitopes, in a longitudinal study of a cohort of patients treated with allo-HCT.

## Supporting Information

Figure S1 Cysteine interferes with T cell recognition of the IE1_290–299_-A*01:01 epitope. Four A*01:01^+^ donors recognize the optimal epitope IE1_290–299_ (TSDACMMTMY). Binding affinity and stability measurements confirmed binding to A*01:01, but IE1_290–299_-A*01:01 tetramer did not stain the CD8^+^ T cells. Instead HLA-matched allo-presentation was used to validate HLA-restriction. In A) *in vitro* cultured PBMCs from donor 14 were analyzed by ICS for CD8^+^ T cell recognition of IE1_290–299_-pulsed autologous DCs (left panel), allogeneic DCs matched for A*01:01 only (middle panel), and allogeneic DCs with no HLA class I match (right panel). In B) three donors were analyzed by *ex vivo* ELISpot for recognition of the wild type epitope, two variants with the internal C substituted for an A (TSDAAMMTMY) or an S (TSDASMMTMY), and an N-terminally truncated 9mer version of the epitope. In C) PBMCs from donor 41 were stained *ex vivo* with A*01:01 tetramers of either the wild type epitope (left) the C→A substituted epitope (middle) or the C→S substituted epitope (right). The plots show gated CD3^+^ cells, and the frequency of tetramer-positive CD8^+^ T cells is indicated.
(EPS)

Table S1 **Identified IE1-specific CD4^+^ T cell epitopes.** The high affinity binding and shared HLA class II molecules within responding donors are underlined. ^a^: Response includes a CD8^+^ T cell epitope. ^b^: Predicted affinity. NB: non binder; only affinity measurements better (i.e. lower) than 1000 nM are shown, peptides binding with an affinity above this threshold are indicated as NB.
(EPS)

Table S2 **Identified IE2-specific CD4^+^ T cell epitopes.** The high affinity binding and shared HLA class II molecules within responding donors are underlined. ^a^: Predicted affinity. ^b^: A subpopulation of T cells could also be stained with an IE2_356–370_-HLA-DRB3*01:01 tetramer. This phenomenon was not observed in donor 14, 19, or 23 (donor 41 not done). ^c^: T cell populations that could be labeled with IE2_408–422_-DRB1*07:01 or IE2_408–422_-DRB1*15:01 were detected. ^d^: Staining with HLA class II tetramer was found negative. NB: non binder; only affinity measurements better (i.e. lower) than 1000 nM are shown, peptides binding with an affinity above this threshold are indicated as NB.
(EPS)

File S1 **A discussion of some of the results, which gave rise to redefinitions of previously published epitopes in terms of peptide-length and/or HLA restriction.**
(DOCX)
