# Supplementary material for: Identification and HLA-Tetramer-Validation of Human CD4+ and CD8+ T Cell Responses against HCMV Proteins IE1 and IE2
Source: PLoS One. 2014 Apr 23;9(4):e94892. doi: 10.1371/journal.pone.0094892 (PMC3997423; doi:10.1371/journal.pone.0094892)
Supplement: File S1 — A discussion of some of the results, which gave rise to redefinitions of previously published epitopes in terms of peptide-length and/or HLA restriction. (DOCX) [file pone.0094892.s004.docx]

**Supporting Information File S1:**

Some of our results lead to a redefinition of the epitope length of already published CD8^+^ T cell epitopes:

a) The IE1_88-95_ epitope was recognized in four donors that only had one allele in common: HLA-C*06:02. The one amino acid longer variant, IE1_87-95_, had previously been reported to be a C*06:02 restricted epitope[56]. We analyzed the binding affinity and stability of both size variants. The short variant, IE1_88-95_-C*06:02 complex is very stable (t½=8.8h), whereas the long variant IE1_87-95_ did not bind to C*06:02 (data not shown). Tetramer labeling validated the IE1_88-95_-HLA-C*06:02 epitope and restriction in all four responding donors.

b) We identified an A*68:01-restricted response against the 9mer IE1_33-41_ epitope. This epitope had been reported as a 10mer elongated N-terminally, IE1_32-41_[52]. Both the 9mer and the 10mer were predicted to be strong binders of A*68:01 and bound to A*68:01, but the 9mer variant, IE1_33-41_ generates a 40 times more stable complex with A*68:01 (t_½_=57h) than the 10mer variant IE1_32-41_ (t_½_=1.4h). Both tetramers could be produced, but only the short variant, IE1_33-41_-A*68:01, was able to stain the CD8^+^ T cells.

Other results lead to a redefinition of the HLA restriction of already published CD8^+^ T cell epitopes.

a) The IE1_309-317_ was recognized in five donors that only had one allele in common: HLA-C*07:02. Three of the responding donors also expressed B*07:02, which has previously been reported as restriction element for this epitope[58]. However, IE1_309-317_ did not bind to B*07:02, but it bound with high stability (t_½_=12.6h) to C*07:02, and IE1_309-317_-C*07:02 tetramer staining confirmed this specificity and restriction. B*07:02 exhibits strong linkage disequilibrium with C*07:02[[1](#_ENREF_1)], which readily explains the erroneous assignment of the epitope restriction to B*07:02. In fact, in the report by Kern et al., all four donors that responded to IE1_309-317_ were also Cw7^+^[58]. After this work was concluded, Ameres et al.[61] have also demonstrated that the IE1_309-317_-epitope is HLA-C*07:02-restricted, rather than HLA-B*07:02-restricted.

b) The IE1_354-363_ was recognized in two donors that had HLA- A*11:01, -B*13:02, and -C*06:02 in common. It has previously been published that the IE1_354-363_ epitope is presented by HLA-A*02:01[60]. However, we did not see this response in any of the four A*02:01^+^ donors. The binding assay, however, showed that HLA-B*13:02, but not HLA- A*11:01 or -C*06:02, was able to bind the epitope. Subsequent IE1_354-363_-B*13:02 tetramer labeling validated this epitope and restriction in both responding donors.

Yet other results have demonstrated how synthetic peptides containing cysteines may be difficult to detect:

The 10mer epitope, IE1_290-299_, was recognized in about half of the A*01:01^+^ donors (four out of nine). A 9mer epitope, IE1_291-299_, truncated one amino acid N-terminally has previously been reported[57]. However, only the 10mer and not the 9mer elicited a cytokine response in our donors (Figure S1B). Both peptides bind to A*01:01, however, the 10mer binds with a 30-fold higher affinity and furthermore achieves a 10-fold higher stability. IE1_290-299_-A*01:01 tetramers were produced to validate the epitope and HLA-restriction, but no specific CD8^+^ T cell staining could be detected. This was surprising given that A*01:01 was the only HLA molecule shared between the four responding donors. In an alternative attempt to validate A*01:01 as the restriction element, we used HLA class I allele matched allo-presentation. Indeed, IE1_290-299_ pulsed target cells from the donor itself and from a donor sharing only A*01:01, but not from a donor without A*01:01, were able to stimulate the specific CD8^+^ T cells (Figure S1A). This confirmed A*01:01 as the restriction element, but left the enigma of why the IE1_290-299_-A*01:01 tetramers failed to stain relevant CD8^+^ T cells. We hypothesized that the centrally positioned cysteine (TSDACMMTMY) could be modified in ways (e.g. through disulphide bond formation) that could interfere with T cell receptor (TcR) interaction. We therefore substituted the cysteine for an alanine or a serine, which bound to A*01:01 with approximately the same affinity and stability as the original epitope (data not shown). Both alanine and serine substitution variants stimulated CD8^+^ T cell specific IFN-γ response comparable to the wild type epitope (Figure S1B). A*01:01 tetramers with both substitution variants of the epitope stained the specific CD8^+^ T cells in all four responding donors (exemplified in Figure S1C). Thus, the cysteine in the peptide used for tetramer formation must be modified in a way that interferes with the TcR recognition, but not with A*01:01 binding. This is a technical issue, which should be kept in mind when evaluating tetramers that have been generated with cysteine-containing peptides.

1. Soen Y, Chen DS, Kraft DL, Davis MM, Brown PO (2003) Detection and characterization of cellular immune responses using peptide-MHC microarrays. PLoS Biol 1: E65.
